# Supplementary material for: Covalently Immobilized Battacin Lipopeptide Gels with Activity against Bacterial Biofilms
Source: Molecules. 2020 Dec 15;25(24):5945. doi: 10.3390/molecules25245945 (PMC7765475; doi:10.3390/molecules25245945)
Supplement: Supplementary file 1 [file molecules-25-05945-s001.pdf]

Supporting Information

Covalently Immobilised Battacin Lipopeptide Gels with Activity against Bacterial Biofilms

Gayan Heruka DeZoysa [a], Kelvin Wang [b], Jun Lu [b], Yacine Hremar[c] and Vijayalekshmi Sarojini[a,d*]

[a] School of Chemical Sciences and the Centre for Green Chemical Science, The University of Auckland, , Auckland- 1142, New Zealand

[b] School of Science, Auckland University of Technology, 34 St Paul Street, Auckland, New Zealand

[c] Department of Biotechnology and Food Engineering, Guangdong Technion Israel Institute of Technology, Shantou, 515063, Guangdong, China

[d] The MacDiarmid Institute for Advanced Materials and Nanotechnology, Wellington 6140, New Zealand

*Author for correspondence

Vijayalekshmi Sarojini

Phone: + 64 9 9233387

E-mail: v.sarojini@auckland.ac.nz

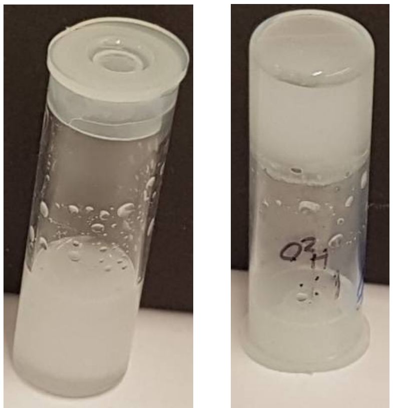

Figure S1: Representative Photograph of peptide-PEG precursors dissolved in water before (left) and after (right) UV photopolymerisation.

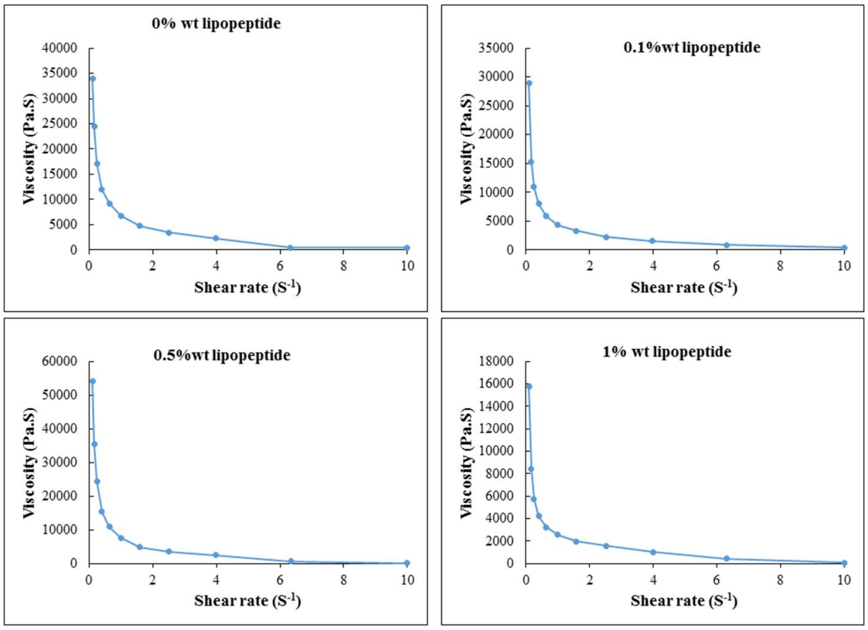

Figure S2: Graph showing results from the sheer thinning experiment of the hydrogel samples containing different concentrations of the lipopeptide prepared in methanol.

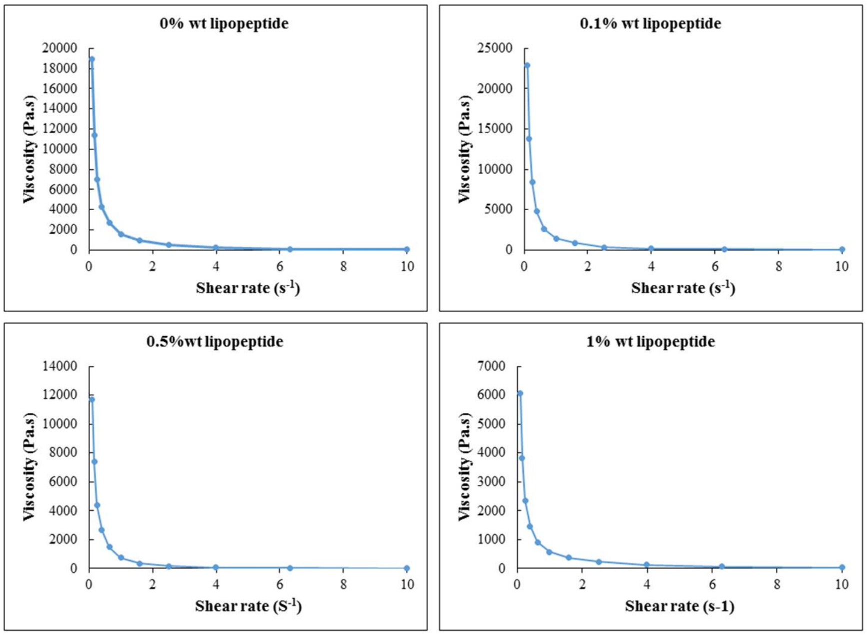

Figure S3: Graph showing results from the sheer thinning experiment of the hydrogel samples containing different concentrations of the lipopeptide prepared in water.

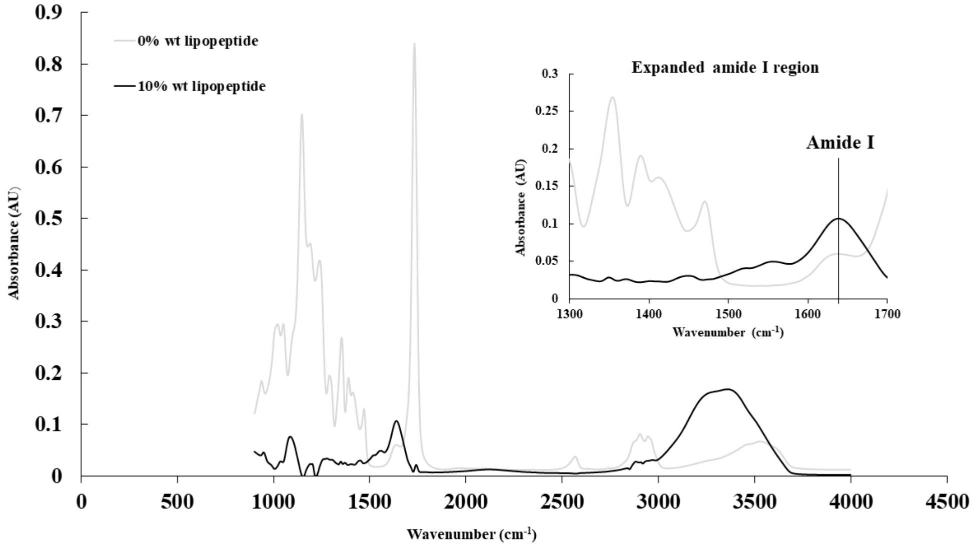

Figure S4: Full FT-IR spectrum of the PEG hydrogels prepared in water in the absence and presence of the lipopeptide

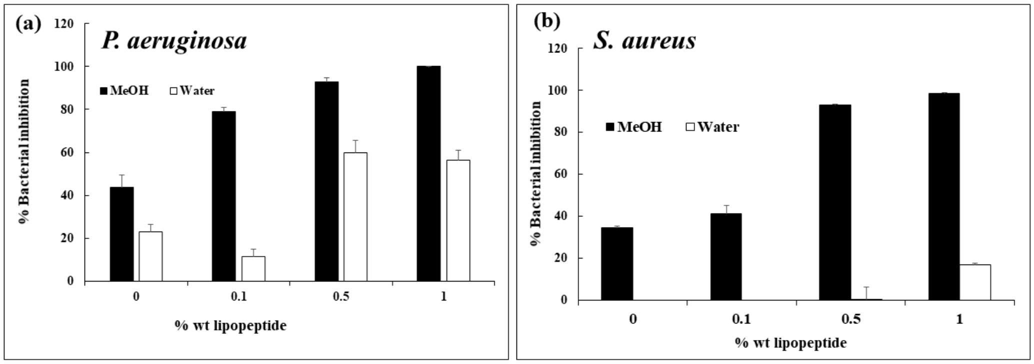

Figure S5: The antibacterial activity of lipopeptide gels against bacterial cells released by the seven days old mature biofilms of (a) *P. aeruginosa* and (b) *S. aureus*.

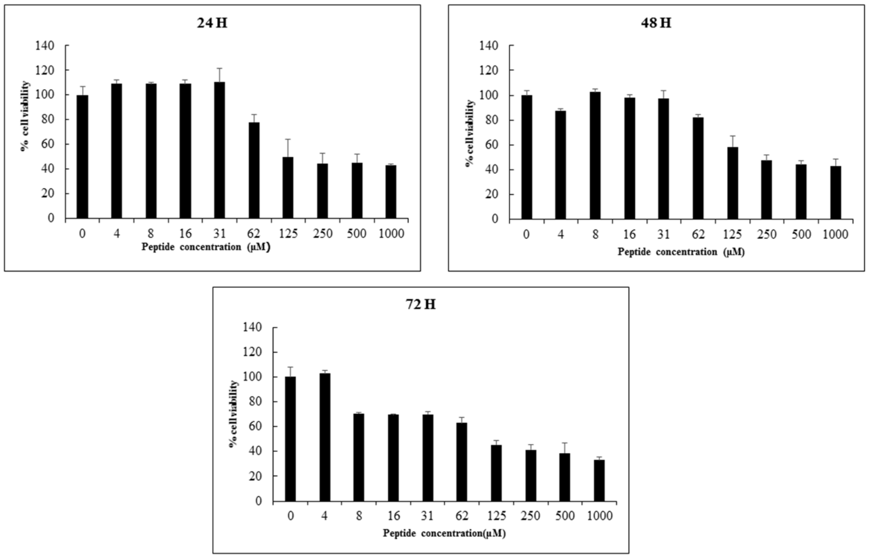

Figure S6: MTT- cell viability assay of human dermal fibroblasts treated with different concentrations of the lipopeptide for 24, 48 and 72 hours.
